# Implementation of KDIGO CKD Screening and Its Prognostic Implications in Community-Dwelling Adults Aged 75 Years or Older: A Population-Based Cohort Study

**DOI:** 10.3390/jcm15166151

**Published:** 2026-08-07

**Authors:** María Isabel Uriarte-Ayestarán, Alicia Gutiérrez-Misis, María Victoria Castell-Alcalá, José M. Mostaza, Carlos Lahoz, Paula Lorenzana-Honorato, Francisco Javier San Andrés-Rebollo, Juan Cárdenas-Valladolid, Pilar Vich-Pérez, Paula Regueiro-Toribio, Miguel Ángel Salinero-Fort

**Affiliations:** 1 Facultad de Medicina, Universidad Autónoma, 28029 Madrid, Spain; alicia.gutierrezm@uam.es (A.G.-M.); mariavictoria.castell@salud.madrid.org (M.V.C.-A.); paula.lorenzana@salud.madrid.org (P.L.-H.); 2 Centro de Salud Ensanche de Vallecas, 28051 Madrid, Spain; 3 Frailty, Multimorbidity Patterns and Mortality in the Elderly Population Residing in the Community-Hospital La Paz Institute for Health Research, IdiPAZ, 28046 Madrid, Spain; josemaria.mostaza@salud.madrid.org (J.M.M.); carlos.lahoz@salud.madrid.org (C.L.); fcojavier.san@salud.madrid.org (F.J.S.A.-R.); juan.cardenas@salud.madrid.org (J.C.-V.); pilar.vich@salud.madrid.org (P.V.-P.); paula.regueiro@salud.madrid.org (P.R.-T.); 4 Centro de Salud Dr. Castroviejo, 28035 Madrid, Spain; 5 Hospital La Paz-Carlos III, 28046 Madrid, Spain; 6 Centro de Salud Baviera, 28028 Madrid, Spain; 7 Centro de Salud Las Calesas, 28026 Madrid, Spain; 8 Foundation for Biomedical Research and Innovation in Primary Care (FIIBAP), 28003 Madrid, Spain; 9 Faculty of Nursing, Universidad Alfonso X el Sabio, 28691 Madrid, Spain; 10 Dirección General de Investigación y Docencia, Consejería de Sanidad de la Comunidad de Madrid, 28013 Madrid, Spain; 11 Centro de Salud Los Alpes, 28022 Madrid, Spain; 12 Centro de Salud Mar Báltico, 28033 Madrid, Spain

**Keywords:** chronic kidney disease, follow-up studies, albuminuria, estimated glomerular filtration rate, all-cause mortality, aged

## Abstract

**Background**: Chronic kidney disease (CKD) is highly prevalent in older adults, yet distinguishing pathological CKD from age-related decline in kidney function remains challenging. KDIGO guidelines recommend combined assessment of estimated glomerular filtration rate (eGFR) and urinary albumin-to-creatinine ratio (uACR), but implementation in primary care is uncertain. We evaluated CKD screening patterns, KDIGO risk categories, and the prognostic value of eGFR and albuminuria for all-cause mortality in adults aged ≥75 years. **Methods**: We conducted a retrospective population-based cohort study of 587,603 community-dwelling adults aged ≥75 years using Primary Care electronic records. Complete CKD screening was defined as at least two eGFR and two uACR measurements ≥ 3 months apart during 2015–2019. CKD prevalence, KDIGO risk categories, and 40-month all-cause mortality were assessed. Multivariable Cox regression models evaluated the independent and joint associations of eGFR and albuminuria with mortality. **Results**: Only 19.0% of participants underwent complete KDIGO-recommended screening, mainly because of limited albuminuria testing. Among screened individuals, CKD prevalence was 25.7%, with over half classified as high or very high KDIGO risk. Mortality increased progressively with declining eGFR, increasing albuminuria, and worsening KDIGO risk. Macroalbuminuria (HR 1.87, 95% CI 1.75–2.00) and eGFR < 30 mL/min/1.73 m^2^ (HR 1.91, 95% CI 1.79–2.04) were independently associated with mortality. **Conclusions**: Complete KDIGO-recommended screening was performed in only one in five adults aged ≥75 years, identifying a major implementation gap in primary care. Albuminuria provided prognostic information beyond eGFR, improving identification of older adults at highest risk of death. These findings support systematic combined eGFR–uACR assessment to improve risk stratification, guide kidney-protective management, and inform healthcare planning for ageing populations.

## 1. Introduction

Chronic kidney disease (CKD) is a prevalent condition worldwide and a major contributor to morbidity, mortality, and health-care costs, particularly those related to cardiovascular disease. An estimated 850 million people are affected globally, with prevalence ranging from 8% to 16% [1]. CKD-related mortality continues to increase, and the CKD is projected to become the fifth leading cause of death globally by 2040 [2].

The burden of CKD continues to raise, driven primarily by population aging and the increasing prevalence of cardiovascular risk factors, notably type 2 diabetes mellitus, obesity, and hypertension, which remain the leading causes [3]. Despite its high prevalence, CKD is frequently underrecognized; epidemiologic studies indicate that up to 85% of older adults with CKD are unaware of their condition, which delays the implementation of preventive and therapeutic interventions [4].

In Spain, population-based studies such as ENRICA and IBERICAN have reported CKD prevalence rates ranging from approximately 14 to 15% in the general population [5,6]. Prevalence increases sharply with age, exceeding 20% among individuals older than 60 years and reaching up to 40% among octogenarians and those with coexisting cardiovascular disease [7]. Projections suggest that CKD may become one of the leading causes of death in Spain in the coming decades, with important clinical and socioeconomic consequences [8].

Early detection of CKD is essential to delay disease progression and reduce associated morbidity and mortality. Primary care plays a central role in identifying individuals at increased risk through systematic assessment of the estimated glomerular filtration rate (eGFR) and the urinary albumin-to-creatinine ratio (uACR), particularly among older adults, who have a high burden of comorbidities and cardiovascular risk [9,10].

According to Kidney Disease: Improving Global Outcomes (KDIGO) guidelines, CKD is defined by the presence of structural or functional kidney abnormalities persisting for more than three months, irrespective of etiology. Diagnosis is based on an eGFR < 60 mL/min/1.73 m^2^ and/or markers of kidney damage, including uACR ≥ 30 mg/g, abnormal urinary sediment, or structural abnormalities, confirmed on at least two measurements. CKD is classified according to eGFR categories (G1–G5), and albuminuria categories (A1–A3), based on either the albumin excretion rate or the uACR. This combined framework provides robust stratification of the risk of kidney disease progression, cardiovascular events and both all-cause and cardiovascular mortality, as demonstrated across at least 49 cohort studies [11].

Despite this established framework, important uncertainties remain regarding the relative prognostic contribution of its two core components, uACR and eGFR, in very old adults. Although both markers predict adverse outcomes and mortality in the general population, few studies have directly compared their associations with all-cause mortality in individuals aged 75 years or older, a group in which age-related decline in kidney function may reduce the discriminative capacity of the eGFR. To our knowledge, this question has not been adequately addressed in a well-defined elderly cohort, leaving an important gap in risk stratification for this rapidly growing segment of the population.

Distinguishing physiological renal ageing from pathological chronic kidney disease is particularly challenging in very old adults. Glomerular filtration rate declines progressively with age due to structural and functional changes in the kidney, and this reduction does not necessarily indicate active kidney disease. As highlighted by Epstein’s seminal review on renal ageing [12], age-related GFR decline may reflect normal senescence rather than clinically relevant renal injury. In this context, albuminuria provides complementary information that helps differentiate physiological ageing from pathological CKD, as it captures glomerular and tubular damage not explained by ageing alone. Understanding this distinction is essential for accurate CKD classification and risk stratification in individuals aged ≥75 years.

Furthermore, our study was conducted exclusively in individuals aged 75 years or older and represents one of the largest contemporary series reported to date outside of meta-analyses. In addition, no previous study has evaluated the prognostic impact of renal function decline and albuminuria in Mediterranean population, where demographic structure, comorbidity patterns, and lifestyle factors differ from those of Northern and Central European countries. These differences may influence mortality rates stratified by age and sex, underscoring the need for region-specific evidence.

This study has four main objectives. First, we aimed to estimate the proportion of individuals aged 75 years or older in January 2020 who underwent complete CKD screening during the preceding five years (2015–2019). Second, we aimed to determine the prevalence of CKD detected through screening and to describe its prognosis stratification by sex. Third, we evaluated 40-month mortality (2020–2023) according to CKD stage and analyzed mortality rates stratified by sex and age group. Finally, we aimed to compare the relative impact of the uACR and eGFR on all-cause mortality in this older population.

## 2. Material and Methods

### 2.1. Study Design and Participants

This study was a retrospective, longitudinal, population-based analysis nested within the Aged-Madrid cohort. The cohort profile has been described in detail elsewhere. Briefly, it comprises all individuals aged 75 years or older who were alive and residing in the Community of Madrid on 1 January 2020, which was used as the index date. To characterize kidney function and its clinical context, we examined a five-year preindex period from 1 January 2015, to 31 December 2019, during which baseline laboratory, clinical, and anthropometric data were collected from routinely recorded health records.

The source population included 587,603 older adults covered by the Spanish National Health System, representing 98.6% of all residents aged ≥75 years in the Madrid region in 2020 (595,652 individuals). Among them, 111,557 individuals had undergone complete CKD screening and constituted the analytic subgroup for CKD ascertainment according to KDIGO criteria [11].

### 2.2. Data Source

Data were obtained from the same sources used to construct the Aged-Madrid cohort [13]. Primary care electronic medical records (EMRs) provide information on diagnoses, risk factors, lifestyle variables, and laboratory tests, and these data sources have been previously validated for major chronic conditions [14,15,16,17]. Hospital discharge records (CMBD) were used to confirm cardiovascular and renal diagnoses. Laboratory information systems supplied serum creatinine, eGFR, and uACR values, while pharmacy dispensing records from the Official College of Pharmacists of Madrid captured medication exposure. Mortality data were obtained through linkage with the National Death Index (INDEF). All datasets were linked using anonymized unique identifiers and integrated into a single curated database.

### 2.3. Study Variables

#### Definition of Complete CKD Screening, CKD Diagnosis, and KDIGO Risk Classification

Kidney function was assessed using the CKD-EPI equation for eGFR and the urinary albumin-to-creatinine ratio (uACR), in accordance with KDIGO recommendations. Complete CKD screening was defined as having at least two eGFR and two uACR measurements obtained at least 3 months apart during the five-year preindex period (2015–2019), thereby ensuring assessment of chronicity and reducing misclassification due to transient changes in kidney function or albuminuria, consistent with previous epidemiological studies that used repeated measurements to confirm CKD [18,19].

Among individuals meeting the complete-screening definition, CKD was diagnosed when persistent kidney abnormality was documented across repeated measurements. Specifically, CKD was considered present if at least one KDIGO marker of kidney damage, either reduced eGFR or elevated uACR, was abnormal on both determinations, regardless of whether the same marker was altered on each occasion. Accordingly, CKD was diagnosed under any of the following conditions: (a) both eGFR values were <60 mL/min/1.73 m^2^; (b) both uACR values were ≥30 mg/g; or (c) both markers were abnormal on both determinations.

Participants with complete screening were classified according to the KDIGO heatmap risk categories. eGFR was categorized as G1 (≥90 mL/min/1.73 m^2^), G2 (60–89 mL/min/1.73 m^2^), G3a (45–59 mL/min/1.73 m^2^), G3b (30–44 mL/min/1.73 m^2^), G4 (15–29 mL/min/1.73 m^2^), and G5 (<15 mL/min/1.73 m^2^). Albuminuria was categorized as A1 (<30 mg/g), A2 (30–300 mg/g), and A3 (>300 mg/g). KDIGO risk strata (low, moderate, high, and very high risk) were assigned using the most recent eGFR and uACR values recorded during the preindex period.

Comorbidities present before the index date were identified using ICD10, and ICPC2 codes, including hypertension, dyslipidemia, obesity, type 1 and type 2 diabetes, ischemic heart disease, stroke, heart failure, peripheral arterial disease, and previously diagnosed CKD. Socioeconomic status was approximated using the MEDEA deprivation index, an area-level measure based on small geographic units that incorporates neighborhood unemployment, low educational attainment, manual employment, and temporary employment. Higher scores indicate greater deprivation.

### 2.4. Data Processing and Analysis

#### 2.4.1. Data Curation

The data quality procedures mirrored those applied in the Aged-Madrid cohort. Implausible laboratory or blood pressure values were reviewed and corrected when possible; otherwise, they were excluded. Duplicate records were removed only when date of birth, sex, and multiple identical laboratory values coincided.

#### 2.4.2. Missing Data

Several variables in the Aged-Madrid cohort contained missing values, as previously described in the original cohort publication [13]. In the present analysis, missingness was restricted to functional status variables, including activities of daily living and instrumental activities of daily living, which were available for 59.4% of individuals with complete CKD screening. These variables were not required for CKD classification, KDIGO stratification, or mortality analyses. Therefore, they did not affect the main exposure or outcome variables. No imputation was performed because functional status is a clinically complex construct that cannot be reliably inferred from the available covariates, and imputation would risk introducing non-plausible values and model misclassification in this older population.

#### 2.4.3. Statistical Analysis

Continuous variables were summarized as means with standard deviations or medians with interquartile ranges, as appropriate, and categorical variables were expressed as counts and percentages. Group comparisons were performed using *t*-tests, ANOVA, the Mann–Whitney U test, the Kruskal–Wallis test, the chi-square test, or Fisher’s exact test, as appropriate.

The prevalence of KDIGO-defined CKD was estimated with 95% confidence intervals and stratified by sex. For descriptive purposes, we also generated a cross-sectional contingency table based on the most recent eGFR and uACR measurements, which does not account for persistence and therefore reflects point-in-time kidney function rather than confirmed CKD.

Mortality rates were calculated as the number of deaths divided by the total person-years of follow-up and expressed per 1000 person-years. Person-time was accrued from baseline until death or censoring at the end of follow-up. Rates were estimated with 95% confidence intervals assuming a Poisson distribution and were stratified by CKD category, sex, and age group.

Kaplan–Meier survival curves were generated for descriptive crude analyses of the uACR, eGFR, and combined KDIGO categories of eGFR and uACR. Additional Kaplan–Meier curves stratified by sex were used to visually compare survival patterns across kidney function and albuminuria strata.

To assess the prognostic relevance of both eGFR and uACR categories, we fitted Cox proportional hazards models to estimate their independent associations with all-cause mortality. Models were adjusted for age, sex, and a comprehensive set of potential confounders, including diabetes mellitus, hypertension, obesity, cancer, myocardial infarction, stroke, and the use of antiplatelet agents, statins, renin–angiotensin–aldosterone system (RAAS) inhibitors, dipeptidyl peptidase-4 (DPP-4) inhibitors, and sodium–glucose cotransporter-2 (SGLT2) inhibitors. To assess the robustness of the findings, we performed a sensitivity analysis excluding patients who died during the first year of the COVID-19 pandemic, given that this period may have introduced additional variability and potential distortion in the estimates.

We additionally constructed a joint eGFR–uACR risk matrix by incorporating cross-product interaction terms between eGFR and uACR categories into the Cox proportional hazards model, using eGFR ≥ 60 mL/min/1.73 m^2^ and uACR < 30 mg/g as the reference category. This matrix summarizes the combined association of reduced eGFR and elevated albuminuria with mortality across KDIGO-based risk strata, whereas the conventional Cox model estimates the independent association of each marker after adjustment for confounders.

Data were processed and analyzed using SPSS, version 26.0 (SPSS Inc., Chicago, IL, USA), and R, version 4.4.0 (R Foundation for Statistical Computing, Vienna, Austria).

## 3. Results

**Figure 1:**
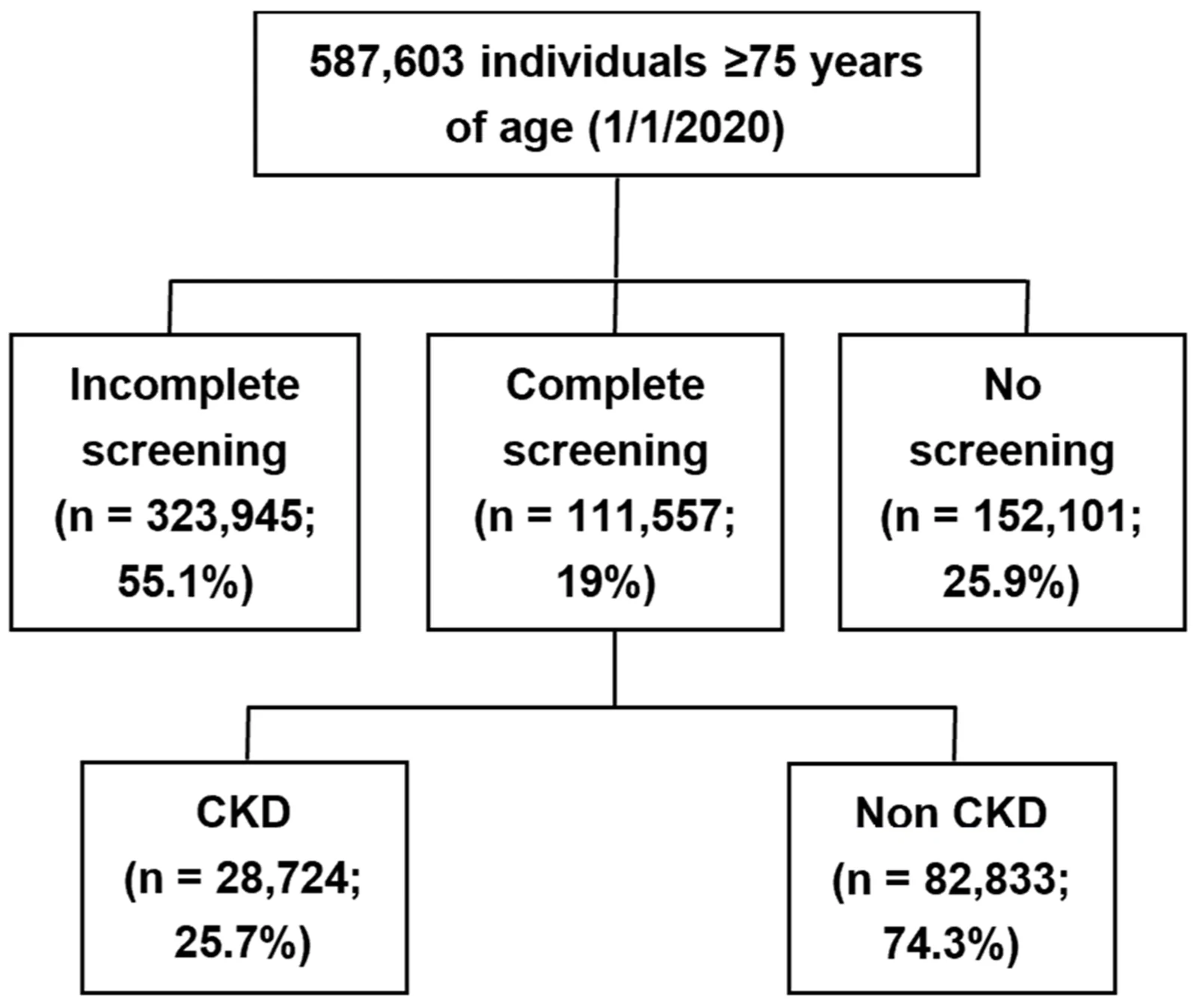
Flowchart of CKD Screening Status and CKD Detection in Adults ≥ 75 Years (*n* = 587,603).

The Aged-Madrid cohort included 587,603 individuals. On the index date, 111,557 participants (19.0%) had ≥2 eGFR and ≥2 uACR measurements recorded between 2015 and 2019 (Figure 1). Among them, 65,614 (58.8%) were women and 45,943 (41.2%) were men, with a mean age of 82.6 years (SD 5.3). The prevalence of hypertension was 82%, diabetes 46.6%, and the remaining comorbidities are shown in Table 1.

### 3.1. CKD Prevalence

Among the 111,557 individuals with complete CKD screening, 28,724 met KDIGO criteria for CKD, corresponding to a prevalence of 25.7% (95% CI, 25.4–26.0%). As shown in Table 2, 20,442 individuals (18.3%) had eGFR < 60 mL/min/1.73 m^2^, and 12,733 (11.4%) had albuminuria (uACR ≥ 30 mg/g). Concurrent reduction on eGFR and albuminuria was present in 4451 individuals (4.0%), whereas 15,991 (14.3%) had isolated eGFR reduction and 8282 (7.4%) had isolated albuminuria.

### 3.2. KDIGO Heatmap Categories According to the Most Recent eGFR and uACR Values Among Individuals with Complete Screening

Among individuals with complete screening, the KDIGO heatmap showed that 10.31% (95% CI, 10.12–10.49) were classified as high risk and 5.75% (95% CI, 5.44–5.70) as very high risk (Table 3). Sex-stratified distributions are provided in Supplementary Tables S1 and S2.

### 3.3. KDIGO Heatmap Categories According to the Most Recent eGFR and uACR Values Among Individuals with CKD (KDIGO Diagnostic Criteria)

Among participants meeting KDIGO diagnostic criteria for CKD, 32.33% (95% CI, 31.79–32.87) were classified as high risk and 21.16% (95% CI, 20.69–32.87) as very high risk (Table 3).

Among the 28,724 individuals aged ≥75 years with a CKD diagnosis, most fell within low-to-moderate KDIGO heatmap categories, predominantly G3a–A1 and G3b–A1. Albuminuria was the main driver of higher risk: categories A2 and A3 shifted patients toward high and very-high risk even when eGFR remained above 45 mL/min/1.73 m^2^. Very-high-risk strata were concentrated in individuals with eGFR < 45 or A3 albuminuria, highlighting substantial heterogeneity in risk despite a uniform CKD diagnosis (Table 4).

Sex-stratified analyses (Table 5 and Table 6) showed clear differences in risk profiles. Men exhibited a more proteinuric phenotype, with higher prevalence of A2 and A3 albuminuria (55.07% and 10.42%, respectively), whereas women predominantly fell into A1 (53.26%). In contrast, women showed a greater proportion of advanced eGFR stages, particularly G4 (8.31% vs. 4.94% in men). As a result, men accumulated more high and very-high KDIGO risk combinations driven by albuminuria, while women were concentrated in moderate-risk strata driven by reduced eGFR but lower albuminuria.

### 3.4. Comorbidity Profile

**Figure 2:**
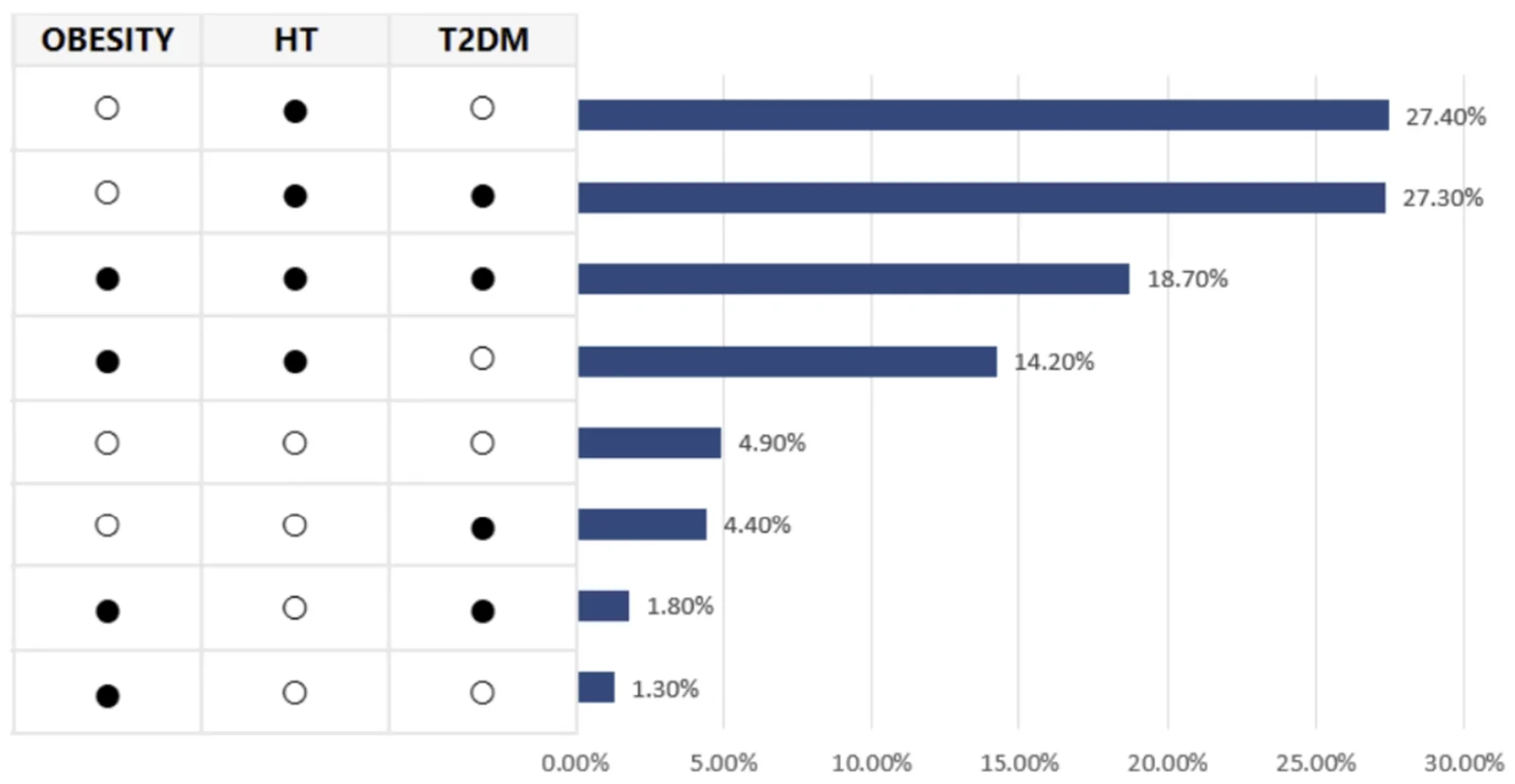
Distribution of Cardiovascular Risk Factor Combinations in Individuals With CKD. HT: hypertension: T2DM: Type 2 Diabetes Mellitus. Solid circle (●) indicates presence of the condition; open circle (○) indicates absence.

**Figure 3:**
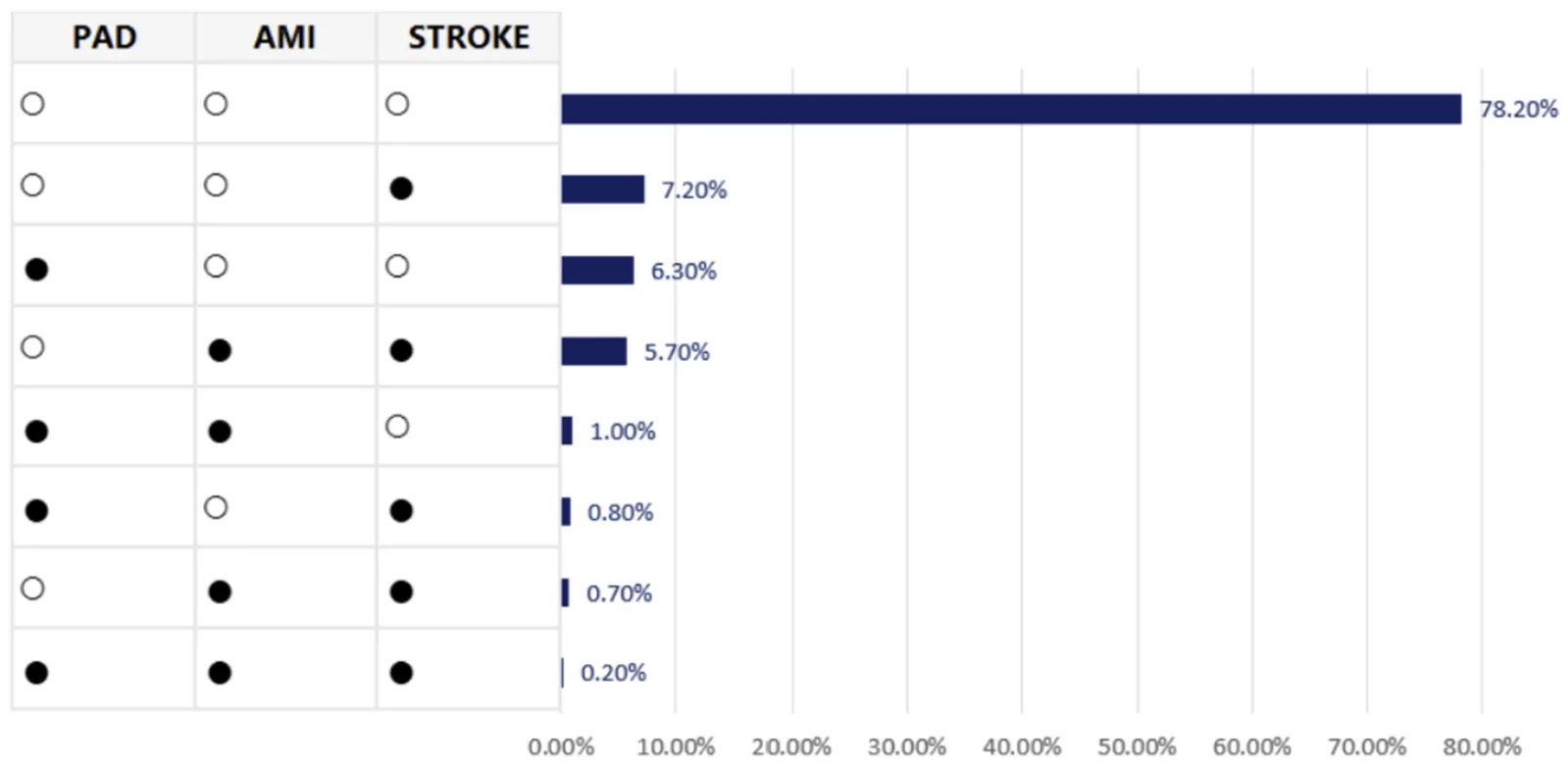
Distribution of Cardiovascular Diseases in Individuals With CKD. PAD: Peripheral Artery Disease; AMI: Acute Myocardial Infarction. Solid circle (●) indicates presence of the condition; open circle (○) indicates absence.

Among individuals with established CKD, cardiometabolic comorbidity was common. Hypertension alone was the most frequent profile (27.40%; 95% CI, 26.89–27.92), closely followed by the combination of type 2 diabetes and hypertension (27.30%; 95% CI, 26.78–27.82). Additional comorbidities and the distribution of established cardiovascular diseases are shown in Figure 2 and Figure 3.

### 3.5. Pharmacologic Therapy

**Figure 4:**
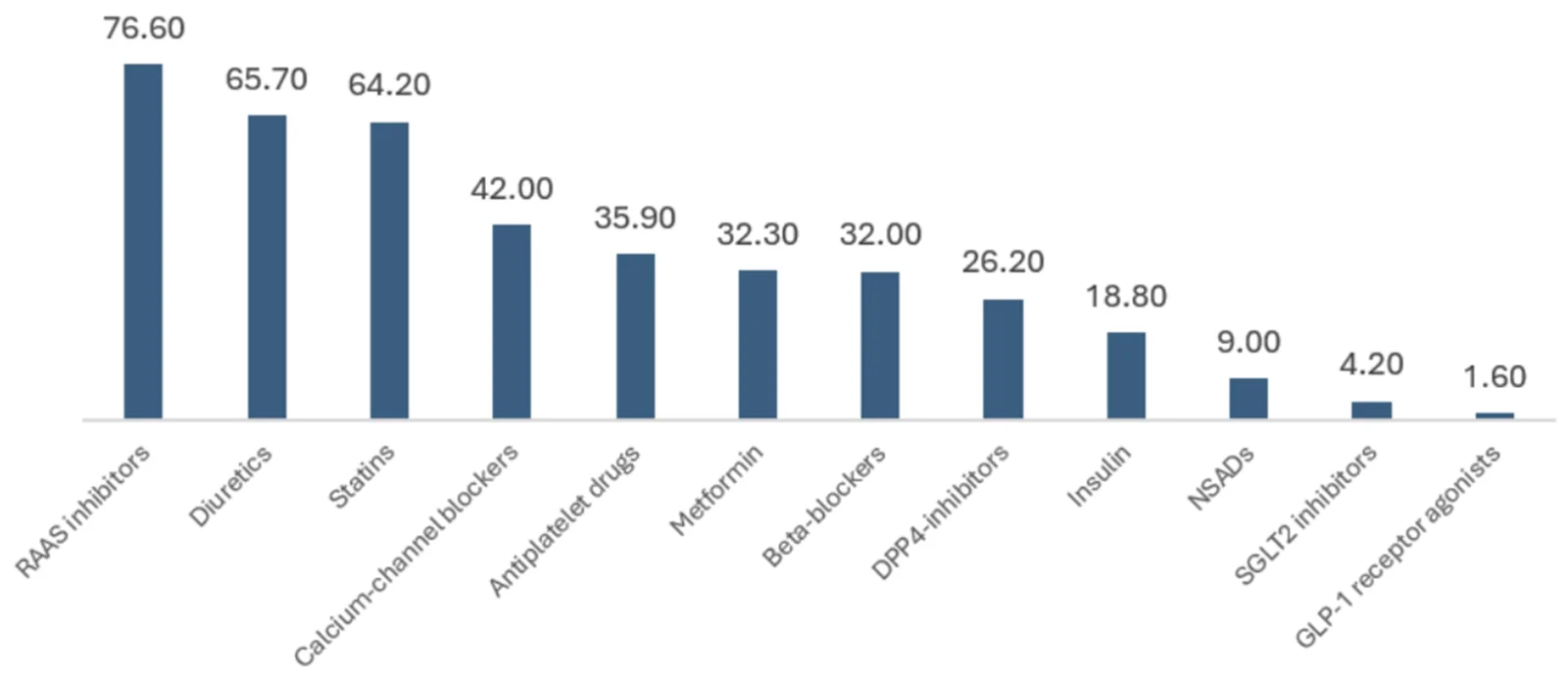
Distribution of Pharmacologic Therapy (%), in patients with CKD. Values represent the relative frequency (%) with respect to the total population with CKD (*n* = 28,724) RAAS inhibitors: Renin–Angiotensin–Aldosterone System inhibitors. DPP-4 inhibitors: dipeptidyl peptidase-4 inhibitors. NSAIDs: Non-steroidal anti-inflammatory drugs. SGLT2 inhibitors: sodium-glucose co-transporter 2 inhibitors. GLP-1 receptor agonists: glucagon-like peptide-1 receptor agonists.

Among participants with CKD, renin–angiotensin–aldosterone system (RAAS) inhibitors were the most frequently used medications (76.60%; 95% CI, 76.10–77.10), followed by diuretics (65.70%; 95% CI, 65.15–66.25), and statins (64.20%; 95% CI, 63.70–64.80). The full distribution of baseline pharmacologic therapy across medication classes is shown in Figure 4.

### 3.6. Mortality Rates

During 40 months of follow-up, 20,915 deaths occurred over 333,529 person-years, yielding an overall crude mortality rate of 62.7 per 1000 person-years (95% CI, 61.9–63.6). Mortality increased progressively with CKD severity, from 49.9 per 1000 person-years in individuals with G1–G2 to 213.9 per 1000 person-years in those with G4–G5.

Sex-specific analyses showed consistently higher mortality in men across all CKD categories, although the relative gradient associated with worsening kidney function was similar in both sexes (Table 7).

Age- and sex-stratified analyses (Table 8) showed a clear stepwise increase in mortality with worsening CKD across all age groups. Among adults aged 75–84 years, mortality rose from 43.2 to 184.2 per 1000 person-years in men and from 23.2 to 115.6 per 1000 person-years in women. In those aged 85–94 years, mortality more than doubled, again showing a graded pattern with CKD severity (men: 136.2 to 315.1; women: 87.1 to 223.9 per 1000 person-years). Among individuals aged ≥95 years, mortality exceeded 300 per 1000 person-years in men across all CKD subgroups, while in women it ranged from 249.4 to 372.0. Reduced eGFR remained a significant predictor of mortality even in the oldest patients.

Overall, advanced CKD (G4–G5) combined with older age identified the groups with the highest absolute mortality.

### 3.7. Mortality and Survival Patterns Across Kidney Function and KDIGO Risk Strata

**Figure 5:**
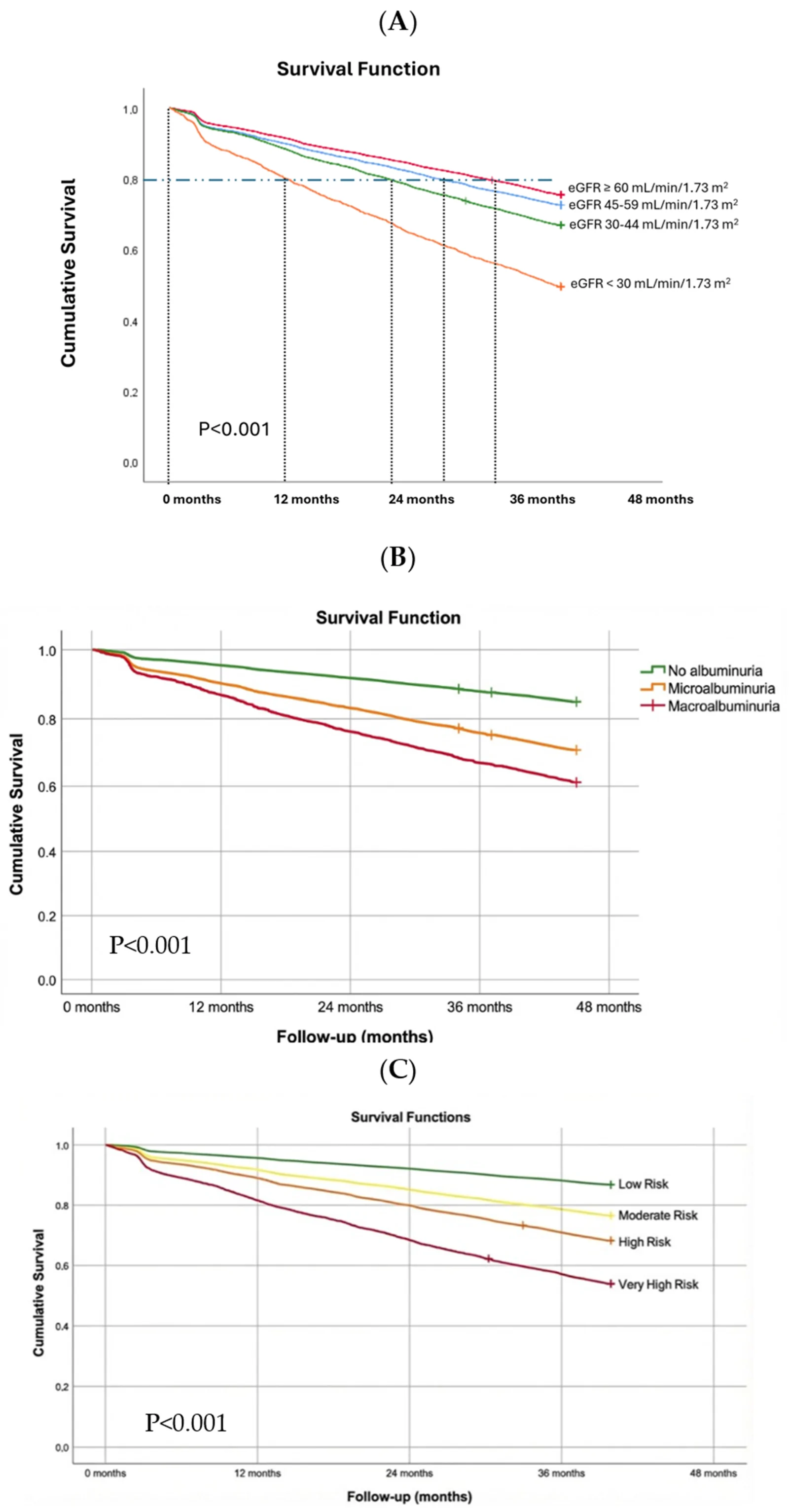
Kaplan–Meier survival analysis across kidney function, albuminuria, and KDIGO risk strata. (**A**) Kaplan–Meier survival curves according to eGFR Category over 40 months of follow-up. eGFR: estimated glomerular filtration rate. (**B**) Kaplan–Meier survival curves according to albuminuria status over 40 months of follow-up. (**C**) Kaplan–Meier survival curves according to KDIGO Risk Category over 40 months of follow-up. The vertical lines represent the projected time at which each eGFR category attains a cumulative survival of 0.80, allowing direct comparison across groups.

Participants with preserved eGFR and normoalbuminuria showed substantially better survival, whereas those with eGFR < 45 mL/min/1.73 m^2^, macroalbuminuria, or high/very-high KDIGO risk exhibited progressively reduced cumulative survival (Figure 5). These gradients were consistent across sexes. Supplementary Figures S1–S3 illustrate additional sex differences; for example, the 80th-percentile survival time in the eGFR 30–44 mL/min/1.73 m^2^ subgroup was 20 months in men and 26 months in women, indicating a six-month survival advantage for women.

### 3.8. Multivariate Analysis

In fully adjusted Cox models, mortality showed a clear graded association with both eGFR and uACR. Compared with individuals with eGFR ≥ 60 mL/min/1.73 m^2^, risk increased from HR 1.419 (95% CI, 1.361–1.480) for eGFR 30–44 mL/min/1.73 m^2^ to HR 1.911 (95% CI, 1.791–2.039) for eGFR < 30 mL/min/1.73 m^2^. Albuminuria was also independently associated with mortality, with a strong severity-related gradient: microalbuminuria was linked to a 54% higher risk (HR 1.542; 95% CI, 1.496–1.590), and macroalbuminuria nearly doubled the risk (HR 1.870; 95% CI, 1.749–2.000) (Table 9). Consistent with these findings, KDIGO CKD risk categories showed a similarly strong stepwise association with mortality in adjusted models (Table 10).

### 3.9. Joint eGFR-uACR Risk Matrix

The interaction model with cross-product terms between eGFR and uACR categories revealed a clear, monotonic gradient of mortality risk across the joint eGFR × uACR matrix (Table 11). Using eGFR ≥ 60 mL/min/1.73 m^2^ and uACR < 30 mg/g as the reference category, HRs increased progressively with both declining eGFR and increasing albuminuria. Moderate albuminuria (uACR 30–299 mg/g) increased mortality risk across all eGFR strata, with HRs ranging from 1.68 (95% CI 1.61–1.75) in individuals with preserved eGFR to 2.90 (95% CI 2.01–4.17) in those with eGFR < 30. Severe albuminuria (uACR ≥ 300 mg/g) showed even stronger associations, reaching HR 3.92 (95% CI 2.28–6.75) in individuals with eGFR < 30. Within each albuminuria category, lower eGFR was consistently associated with higher mortality, and within each eGFR category, higher albuminuria was consistently associated with higher mortality.

Given the COVID-19 pandemic during 2020, a sensitivity analysis excluding deaths occurring in 2020 was performed, yielding similar results. See Supplementary Tables S3 and S4.

## 4. Discussion

In this population-based cohort of adults aged 75 years or older, we evaluated CKD screening patterns, KDIGO risk categories, and 40-month mortality, and assessed the prognostic value of eGFR and uACR in very old adults. Overall, three findings deserve emphasis. First, combined screening with eGFR and albuminuria remained clearly suboptimal in routine practice. Second, albuminuria provided prognostic information beyond eGFR alone. Third, mortality increased progressively across KDIGO risk strata, supporting the clinical relevance of the combined classification in advanced age.

### 4.1. CKD Screening

Only 19% of participants met criteria for complete CKD screening over the pre-index period (2015–2019), despite current KDIGO recommendations emphasizing the combined assessment of eGFR and albuminuria. This gap is clinically relevant because albuminuria testing remains substantially underused in both our cohort and routine care, with large proportions of at-risk individuals not undergoing uACR measurement [20]. Similar deficiencies have been reported in patients with diabetes, in whom annual screening with both markers remains far from universal [21]. Together, these findings indicate a persistent disconnect between guideline recommendations and real-world implementation.

The limited use of albuminuria testing has important implications for case detection and risk stratification. In the ONDAAS study, systematic uACR assesment identified previously unrecognized CKD and reclassified many individuals into high- or very high-risk categories that would have been missed by eGFR alone [10]. Likewise, the PROGRESER cohort showed that albuminuria was the strongest predictor of kidney function decline, whereas diabetes alone did not adequately identify progression risk [22]. These findings reinforce that incomplete screening may delay diagnosis and reduce access to disease-modifying interventions [3,11].

Beyond its role in CKD detection, wider implementation of uACR testing in at-risk individuals is essential for accurate risk stratification and identification of patients who may benefit from evidence-based nephroprotective therapies, as recommended by current KDIGO guidelines [11].

Individuals undergoing complete CKD screening showed a consistently higher cardiometabolic burden, including myocardial infarction (5.4% vs. 4.4%, *p* < 0.001), stroke (6.7% vs. 5.9%, *p* < 0.001), peripheral artery disease (5.9% vs. 4.0%, *p* < 0.001), diabetes mellitus (46.6% vs. 18.8%, *p* < 0.001) and dyslipidemia (64.0% vs. 50.2%, *p* < 0.001). They also exhibited greater use of cardioprotective therapies, such as SGLT2 inhibitors (3.1% vs. 1.1%, *p* < 0.001), RAAS inhibitors (71.3% vs. 52.9%, *p* < 0.001) and antiplatelet agents (31.6% vs. 25.4%, *p* < 0.001). This pattern suggests that CKD screening in practice was mainly triggered by pre-existing high-risk conditions, reflecting an opportunistic rather than systematic approach. Although age alone would also justify screening [23] testing appears to have been prioritized in patients already known to have hypertension, diabetes, or other cardiovascular risk factors. This behavior is consistent with current recommendations favoring targeted screening in high-risk populations, where the yield and clinical impact are greatest [24,25].

By contrast, participants without evidence of CKD screening had lower recorded cardiometabolic burden but higher socioeconomic deprivation and lower use of cardioprotective medications. This is relevant because socioeconomic disadvantage is consistently associated with higher CKD prevalence, faster progression, and worse outcomes [26,27,28]. Since early CKD is often asymptomatic and depends on laboratory testing for detection, limited screening in socially disadvantaged individuals may contribute to underdiagnosis and widening health inequities [27,28,29].

### 4.2. CKD Prevalence and Risk Categories in Individuals with Complete CKD Screening

Among participants with complete CKD screening, CKD prevalence was 25.7%. This estimate is consistent with prior studies in older adults, which have shown that CKD is common in late life. In the Spanish EPIRCE study [30], CKD prevalence among individuals older than 64 years was 21.42%. Similar or higher figures were reported in NHANES [31] and the UK HSE [32], particularly in people aged 75 years or older. These findings reinforce the need for systematic evaluation of kidney disease in older adults.

According to KDIGO heat-map categories [11], 10.31% of participants with complete screening were classified as high risk and 5.75% as very high risk. High-risk CKD was slightly more frequent in women, although the difference was not significant, whereas very high-risk CKD was nearly identical between sexes. This pattern is consistent with prior evidence showing that sex differences in CKD are modest after accounting for age and cardiometabolic burden [33,34]. In older adults, disease severity and multimorbidity may outweigh sex as determinants of risk [34,35].

### 4.3. Treatment Patterns

Use of cardioprotective therapy was greater among individuals who underwent complete screening, particularly for RAAS inhibitors and SGLT2 inhibitors, which were reported solely as part of the observed treatment profile. Particularly noteworthy was the low use of SGLT2 inhibitors, likely related to the timing of baseline prescription (end of 2019). At that point, nephroprotective evidence for these agents was still emerging. The CREDENCE trial (2019) had demonstrated renal benefits in patients with type 2 diabetes and albuminuric CKD, but its findings had not yet translated into widespread use in the broader older CKD population. Subsequent landmark trials, including DAPA-CKD (2020) and EMPA-KIDNEY (2022), later confirmed substantial reductions in CKD progression, cardiovascular events, and mortality across a wide range of patients, including older adults and individuals without diabetes [36,37]. Consequently, current KDIGO 2024 guidelines recommend SGLT2 inhibitors for eligible patients with CKD irrespective of diabetes status [11].

An additional consideration is that the evidence supporting the use of several cardioprotective therapies in very old adults remains limited. Most pivotal randomized clinical trials evaluating RAAS inhibitors included very few participants aged ≥80 years, and current recommendations are therefore largely extrapolated from younger populations. As a result, treatment decisions in this age group require careful individualization, taking into account frailty, multimorbidity, polypharmacy, functional status, and life expectancy, as well as the need for close clinical monitoring. Our observational design was not intended to evaluate treatment efficacy or safety; accordingly, we refrain from making therapeutic recommendations beyond guideline-supported individualized care.

### 4.4. Risk Categories Among Patients with CKD

Among participants with established CKD, the burden of high and very high KDIGO risk categories was slightly higher in men than in women, although the absolute difference was modest. This aligns with previous reports showing sex-related differences in CKD epidemiology, including variations in albuminuria, kidney function, and disease progression across populations [38].

Among 28,724 participants with CKD, multimorbidity was the rule rather than the exception, and cardiometabolic clusters were dominant. CKD with hypertension alone was the most common profile, followed by combined hypertension, and diabetes. These patterns are consistent with the established role of these conditions in accelerating kidney damage and cardiovascular risk. Chronic hyperglycemia and sustained hemodynamic stress promote albuminuria, glomerular injury, and progressive loss of kidney function [39,40]. In addition, CKD is strongly linked to cardiovascular disease, including peripheral artery disease, myocardial infarction, and stroke, underscoring the close kidney–heart interaction [41,42]. This pattern aligns with prior population-based analyses of multimorbidity in CKD [43,44].

### 4.5. Mortality Rates

CKD was associated with a progressive increase in mortality, with the highest rates (per 1000 person-years) observed in advanced stages (G4–G5). This pattern is consistent with the established association between lower eGFR, higher albuminuria, and mortality risk across diverse populations, including older adults [45]. Although mild reductions in eGFR at older ages may reflect age-related physiological changes, our data show significant increases in mortality in the most advanced stages, confirming the value of eGFR-based categorization for identifying high-risk subgroups even among elderly patients [11,46].

Mortality rates were higher in men across CKD strata, particularly in advanced stages. However, the relative increase in mortality with worsening kidney function was similar in both sexes, reinforcing the strong prognostic impact of CKD itself. In the oldest age groups, absolute mortality rates rose steeply in both sexes, while sex differences narrowed, suggesting that frailty and comorbidity may increasingly shape outcomes in late life [35,47].

Over 40 months of follow-up, survival differed markedly according to kidney function and KDIGO risk category. Individuals with preserved kidney function and low KDIGO risk had the best survival, whereas those with eGFR below 45 mL/min/1.73 m^2^, macroalbuminuria, or high KDIGO risk showed progressively worse survival. These findings support the value of the KDIGO framework for risk stratification in very old adults [48].

### 4.6. Mortality Multivariate Adjusted Analysis

Our multivariable analyses confirmed that both reduced eGFR and elevated albuminuria were independently associated with all-cause mortality, even after adjustment for comorbidities and cardioprotective therapies. Compared with preserved kidney function and normoalbuminuria, eGFR < 30 mL/min/1.73 m^2^ was associated with substantially higher mortality, and macroalbuminuria showed a similarly adverse association. These findings reinforce the complementary prognostic value of eGFR and albuminuria in advanced age, consistent with contemporary evidence [49,50].

The joint eGFR–uACR risk matrix derived from our interaction model showed a clear, monotonic gradient of mortality risk across all combinations of kidney filtration and albuminuria, with the highest hazard ratios in individuals presenting both severely reduced eGFR and marked albuminuria. This pattern underscores the prognostic relevance of evaluating kidney filtration and albuminuria jointly rather than independently, particularly in very old adults.

Our findings are broadly consistent with the landmark meta-analysis by Matsushita et al. [45], which demonstrated independent and multiplicative associations of eGFR and albuminuria with mortality across 21 general population cohorts. In that analysis, hazard ratios increased steadily with declining eGFR and rising albuminuria, and the authors reported no statistically significant interaction between the two measures. Consequently, their risk matrix was constructed by combining independent effects rather than estimating cell-specific interaction coefficients.

By incorporating interaction terms between eGFR and uACR, our model captured the combined burden of filtration impairment and albuminuria in very old adults, generating cell-specific hazard ratios that reflect their joint prognostic impact. The combined assessment of eGFR and uACR provided more informative risk stratification than eGFR alone, particularly in the context of age-related declines in GFR that may obscure clinically relevant kidney disease. These findings align with the CKD Prognosis Consortium, which showed that even modest increases in albuminuria are associated with higher mortality across all levels of kidney function [45], identifying a high-risk phenotype not captured by eGFR alone.

Age-related changes in renal function further complicate interpretation. The Baltimore Longitudinal Study of Aging reported an average annual decline in creatinine clearance of 0.75 mL/min/year [51]. In parallel, some authors have suggested that eGFR values of 45–50 mL/min/1.73 m^2^ may be physiological in individuals around 80 years of age [52], despite overlapping with CKD stage 3. This overlap highlights the difficulty of distinguishing physiological aging from clinically meaningful kidney disease, highlighting albuminuria as a key marker of active renal injury.

Recent reviews further emphasize that eGFR and albuminuria are essential not only for identifying kidney damage but also for prognostic stratification, as both reflect systemic cardiovascular risk [48,53]. Reliance on eGFR alone may underestimate risk in older adults and in individuals with cardiometabolic comorbidity, as shown by the CKD Prognosis Consortium [45]. Our results also support that macroalbuminuria is a more direct marker of active glomerular and systemic vascular injury, which may explain its stronger association with mortality than CKD stage 3 based solely on eGFR. This pattern is consistent with geriatric cohorts in which albuminuria repeatedly emerges as a robust prognostic marker [49,50]. Longitudinal evidence likewise shows that sustained albuminuria predicts mortality and CKD progression independently of eGFR, reinforcing its role as a marker of active renal and vascular pathology [54].

The integration of the KDIGO classification system further reinforces its clinical utility. Compared with individuals classified as low risk, those in the moderately increased, high, and very high-risk categories showed progressively higher mortality risk. These findings are consistent with recent studies validating the KDIGO heat map as a predictor of mortality and cardiovascular events across CKD stages [55,56]. Sensitivity analyses excluding deaths in 2020 yielded similar results, indicating that the associations observed were robust and not materially influenced by the COVID-19 pandemic.

### 4.7. Clinical Implications

Our findings have important implications for the organization of CKD care in ageing populations. First, the remarkably low implementation of complete KDIGO-recommended screening, largely driven by the underuse of albuminuria testing, indicates that CKD detection in routine primary care remains predominantly opportunistic rather than systematic. Given the rapid growth of the population aged ≥75 years, wider implementation of combined eGFR and uACR assessment should become a priority for healthcare systems seeking to improve early identification of high-risk individuals.

Second, our results reinforce that albuminuria provides clinically meaningful prognostic information beyond eGFR alone, particularly in very old adults in whom age-related decline in kidney function may complicate the interpretation of reduced eGFR. Routine incorporation of uACR into CKD assessment would therefore improve risk stratification and facilitate identification of patients who may benefit from closer monitoring and evidence-based kidney-protective strategies.

Third, the high burden of multimorbidity observed in this cohort highlights the need for integrated models of care involving primary care physicians, nephrologists, geriatricians, nurses, and community healthcare services. In very old adults, management should extend beyond CKD staging to include comprehensive assessment of frailty, cardiovascular risk, polypharmacy, functional status, and patient preferences, allowing individualized therapeutic decisions.

Finally, although our study was not designed to evaluate treatment effectiveness, the low use of newer kidney-protective therapies observed in this population suggests that implementation of contemporary guideline-directed management remains incomplete. Because evidence from randomized clinical trials in adults aged ≥80 years is still limited, treatment decisions should be individualized, balancing potential renal and cardiovascular benefits against frailty, comorbidity, life expectancy, and tolerability. Improving systematic screening together with multidisciplinary management may help optimize allocation of healthcare resources and reduce CKD-related complications in this rapidly expanding segment of the population.

#### 4.7.1. Weakness

Several limitations should be acknowledged. First, the retrospective design precludes causal inference, and unmeasured confounders may influence the observed associations despite comprehensive multivariable adjustment. Second, the 40-month follow-up may limit long-term prognostic insights, although this period included 20,915 deaths (6.3% annual mortality) in a very old cohort (mean age 82.6 years).

Third, missing data, particularly for the uACR (89.9% incomplete screening), reflect real-world clinical practice but may introduce selection bias toward higher-risk individuals who were screened. To preserve clinical validity, no imputation was performed; denominators were reported transparently, although this approach reduces the precision of prevalence estimates.

Fourth, reliance on CKD-EPI eGFR assumes standardized creatinine calibration across Madrid laboratories, although Spanish primary care systems generally employ traceable assays. Fifth, the exclusion of nursing-home residents and recent immigrants (due to EMR coverage) may limit generalizability beyond community-dwelling older adults.

Sixth, although only 19% of the population had complete KDIGO-recommended screening, this subgroup does not represent a selected sample but rather the proportion of older adults who met the strict diagnostic criteria required to ascertain CKD with full certainty. Our study included the entire population aged ≥75 years in the Madrid region, without exclusions other than those inherent to KDIGO chronicity requirements (≥2 eGFR and ≥2 uACR measurements ≥3 months apart). This design ensures high internal validity; however, external inference should be limited to populations of similar age and screened using comparable methods. Importantly, the CKD prevalence observed in this fully screened subgroup (25.7%) is consistent with other Spanish studies using less stringent definitions, supporting the plausibility and external coherence of our findings. Furthermore, the low uptake of albuminuria testing mirrors patterns reported in other European primary care settings, indicating that incomplete KDIGO screening is a structural feature of routine practice rather than a region-specific phenomenon.

Seventh, the Madrid-specific context may not fully translate to other healthcare systems with different screening practices, comorbidity profiles, or access to cardioprotective therapies. Finally, the low proportion of the population screened between 2015 and 2019 may partly reflect early limitations in ordering albuminuria tests from primary care; therefore, extrapolation of these findings to the current context should be made with caution.

Finally, we were not able to compare mortality rates with an external age- and sex-matched population from the same geographical area. Our cohort was specifically designed to evaluate mortality according to kidney function and KDIGO risk categories among individuals who underwent CKD assessment within the Madrid primary care system, and an appropriate comparator group without CKD was not available within the study design. Future studies incorporating parallel cohorts of older adults without CKD from the same region would allow quantification of the excess mortality attributable to chronic kidney disease and provide additional context for interpreting the prognostic impact observed in our analyses.

#### 4.7.2. Strengths

This study has several notable strengths. First, its population-based design includes 587,603 community-dwelling adults ≥75 years, representing 98.6% coverage of Madrid’s eligible population and minimizing selection bias inherent in clinic-based cohorts. Second, we employed a novel, clinically relevant definition of “complete KDIGO screening” (≥2 eGFR + ≥2 uACR measurements ≥3 months apart), enabling precise quantification of guideline implementation gaps, with only 19% compliance, which is unprecedented in primary care research.

Third, comprehensive data integration combined validated primary care EMRs, hospital discharge records, pharmacy databases, and complete mortality linkage via National Death Index, ensuring robust covariate adjustment (12+ variables including therapies) and eliminating loss-to-follow-up. Fourth, geriatric-specific analyses provide new insights: mortality differences across CKD stages tended to attenuate with advancing age, and reduced eGFR and macroalbuminuria showed comparable magnitudes of risk.

Fifth, a sensitivity analysis excluding patients who died in 2020, during the first waves of the COVID-19 pandemic, yielded results nearly identical to those for the full cohort, indicating that albuminuria, eGFR strata, and KDIGO risk categories remained similarly associated with mortality and supporting the robustness of our findings.

Finally, our primary care perspective, contrasting screened versus unscreened populations, offers actionable evidence for policymakers and clinicians. It demonstrates higher cardiometabolic burden and greater use of cardio-nefroprotective therapies among screened individuals while quantifying socioeconomic disparities in access to kidney screening.

Together, these strengths provide a clinically grounded and policy-relevant basis for interpreting our findings, underscoring how CKD screening practices in primary care shape risk stratification, therapeutic opportunities, and mortality in very old adults.

## 5. Conclusions

In this large population-based cohort of adults aged ≥75 years, we identified a substantial implementation gap in KDIGO-recommended CKD screening, driven primarily by limited albuminuria testing in routine primary care. Only one in five older adults underwent complete assessment with repeated eGFR and uACR measurements, underscoring the need for more systematic and proactive renal evaluation in this rapidly growing segment of the population.

Among individuals who were adequately screened, CKD prevalence was high and KDIGO risk stratification revealed considerable heterogeneity in prognosis. Albuminuria emerged as a key determinant of risk, providing prognostic information beyond that offered by eGFR alone. In very old adults, where age-related decline in eGFR may complicate interpretation, uACR was particularly valuable for distinguishing physiological renal ageing from clinically meaningful kidney damage. Macroalbuminuria and severely reduced eGFR were independently associated with increased mortality, and their combination identified individuals at the highest risk.

From a clinical perspective, these findings reinforce the importance of incorporating uACR into routine CKD evaluation in older adults, as recommended by KDIGO. Systematic combined assessment of eGFR and uACR can improve risk stratification, guide the intensity of follow-up, support decisions regarding nephroprotective therapies, and inform referral pathways to nephrology. Moreover, given the high burden of multimorbidity in this age group, CKD management should be integrated within multidisciplinary care models that include primary care, geriatrics, and nephrology.

Overall, our results highlight the need to strengthen CKD screening practices in primary care and to adopt a more comprehensive, risk-based approach to kidney health in older adults. Implementing combined eGFR–uACR assessment may enhance clinical decision-making, optimize resource allocation, and ultimately improve outcomes in ageing populations.

## Figures and Tables

**Table 1:** Baseline characteristics of older adults (≥75 years) undergoing complete CKD screening in primary care.

	Completed Screening (*n* = 111,557)
Age, years	82.6 (5.3)
Male, sex	45,943 (41.2)
Severe or total dependence, n/N	2478/45,296 (5.5)
Hypertension	91,514 (82.0)
Diabetes Mellitus	51,973 (46.6)
Dyslipidemia	71,438 (64.0)
Dementia	7330 (6.6)
Myocardial infarction	6069 (5.4)
Stroke	7469 (6.7)
PAD	6450 (5.9)
Atrial Fibrillation	18,667 (16.7)
Known CKD	10,465 (9.4)
Cancer	22,109 (19.8)
BMI, ksg/m^2^	29.1 (4.7)
Deprivation index	0.14 (0.91)
Antiplatelet drugs	32,250 (31.6)
Diuretics	57,401 (51.5)
Beta-blockers	26,715 (23.9)
Calcium-channel blockers	33,362 (29.9)
Metformin	38,145 (34.2)
Insulin	11,388 (10.2)
NSAIDs	10,599 (9.5)
DPP-4 inhibitors	20,918 (18.8)
SGLT2 inhibitors	3403 (3.1)
GLP-1 receptor agonists	984 (0.9)
Statins	69,930 (62.7)
RAAS inhibitors	79,564 (71.3)

Notes: Values are presented as mean (standard deviation) for continuous variables and count (percentage) for categorical variables. N is total number of participants with available information. CKD: chronic kidney disease. CKD screening status was categorized as: fully completed (≥2 eGFR and ≥2 uACR measurements separated by ≥3 months), partially completed (only one marker or insufficient repetition), and no evidence of screening (absence of qualifying eGFR and uACR measurements). Asterisks indicate a significant linear trend across screening status categories. BMI: body mass index. PAD: peripheral artery disease. DPP-4 inhibitors: dipeptidyl peptidase-4 inhibitors. SGLT2 inhibitors: sodium-glucose co-transporter 2 inhibitors. GLP-1 receptor agonists: glucagon-like peptide-1 receptor agonists. NSAIDs: Non-steroidal anti-inflammatory drugs. The deprivation index corresponds to the MEDEA deprivation index, an area-level socioeconomic indicator derived from census variables (unemployment, manual employment, temporary employment, low educational attainment). Scores are standardized, with higher values indicating greater deprivation.

**Table 2:** Contingency table of eGFR and albuminuria categories (*n* = 111,557).

eGFR < 60 mL/min/1.73 m^2^	uACR ≥ 30 mg/g	uACR < 30 mg/g	Total
Yes	4451 (4.0%)	15,991 (14.3%)	20,442 (18.3%)
No	8282 (7.4%)	82,833 (74.3%)	91,115 (81.6%)
Total	12,733 (11.4%)	98,824 (88.6%)	111,557 (100%)

Classification was based on at least two eGFR measurements < 60 mL/min/1.73 m^2^ and/or at least two uACR measurements ≥ 30 mg/g, obtained at least three months apart. Values in parentheses represent percentages calculated over the total study population (*n* = 111,557).

**Table 3:** KDIGO heatmap categories among 111,557 adults aged ≥75 years with complete CKD screening, based on the most recent eGFR and uACR values recorded between 2015–2019.

eGFR/uACR	A1	A2	A3	Total
G1	3834 (3.44)	677 (0.61)	49 (0.04)	4560 (4.09)
G2	61,926 (55.51)	12,358 (11.08)	994 (0.89)	75,278 (67.48)
G3a	14,829 (13.29)	4960 (4.45)	602 (0.54)	20,391 (18.28)
G3b	5503 (4.93)	3105 (2.78)	535 (0.48)	9143 (8.20)
G4	894 (0.80)	921 (0.83)	272 (0.24)	2087 (1.87)
G5	15 (0.01)	44 (0.04)	39 (0.03)	98 (0.09)
Total	87,001 (77.99)	22,065 (19.78)	2491 (2.23)	111,557 (100)

Values in parentheses represent the relative frequency (%) with respect to the total population (*N* = 111,557). eGFR: estimated glomerular filtration rate; UACR: urine albumin-to-creatinine ratio. G1 (≥90 mL/min/1.73 m^2^), G2 (60–89), G3a (45–59), G3b (30–44), G4 (15–29), G5 (<15). A1 (<30 mg/g), A2 (30–300 mg/g), A3 (>300 mg/g). Cells are color-coded according to KDIGO risk categories: green = low risk (if no other markers of kidney damage are present), yellow = moderately increased risk, orange = high risk, and red = very high risk.

**Table 4:** KDIGO heatmap categories among 28,724 individuals aged ≥75 years with a CKD diagnosis, based on the most recent eGFR and uACR values recorded between 2015–2019.

eGFR/uACR	A1	A2	A3	Total
G1	0 (0.00)	289 (1.01)	40 (0.14)	329 (1.15)
G2	0 (0.00)	5559 (19.35)	778 (2.71)	6337 (22.06)
G3a	7514 (26.16)	3813 (13.27)	553 (1.93)	11,880 (41.36)
G3b	4656 (16.21)	2931 (10.20)	525 (1.83)	8112 (28.24)
G4	803 (2.80)	901 (3.14)	268 (0.93)	1972 (6.87)
G5	13 (0.05)	43 (0.15)	38 (0.13)	94 (0.33)
Total	12,986 (45.21)	13,536 (47.12)	2202 (7.67)	28,724 (100)

Values in parentheses represent the relative frequency (%) with respect to the total population (*N* = 111,557). eGFR: estimated glomerular filtration rate; uACR: urine albumin-to-creatinine ratio. G1 (≥90 mL/min/1.73 m^2^), G2 (60–89), G3a (45–59), G3b (30–44), G4 (15–29), G5 (<15). A1 (<30 mg/g), A2 (30–300 mg/g), A3 (>300 mg/g). Cells are color-coded according to KDIGO risk categories: green = low risk (if no other markers of kidney damage are present), yellow = moderately increased risk, orange = high risk, and red = very high risk.

**Table 5:** KDIGO heatmap categories among 12,337 men aged ≥75 years with a CKD diagnosis, based on the most recent eGFR and uACR values recorded between 2015–2019.

eGFR/uACR	A1	A2	A3	Total
G1	0 (0.00)	165 (1.34)	16 (0.13)	181 (1.47)
G2	0 (0.00)	3149 (25.52)	503 (4.08)	3652 (29.60)
G3a	2719 (22.04)	1916 (15.53)	337 (2.73)	4972 (40.30)
G3b	1379 (11.18)	1232 (9.99)	282 (2.29)	2893 (23.45)
G4	159 (1.29)	318 (2.58)	133 (1.08)	610 (4.94)
G5	1 (0.01)	14 (0.11)	14 (0.11)	29 (0.24)
Total	4258 (34.51)	6794 (55.07)	1285 (10.42)	12,337 (100)

Values in parentheses represent the relative frequency (%) with respect to the total population (*N* = 111,557). eGFR: estimated glomerular filtration rate; uACR: urine albumin-to-creatinine ratio. G1 (≥90 mL/min/1.73 m^2^), G2 (60–89), G3a (45–59), G3b (30–44), G4 (15–29), G5 (<15). A1 (<30 mg/g), A2 (30–300 mg/g), A3 (>300 mg/g). Cells are color-coded according to KDIGO risk categories: green = low risk (if no other markers of kidney damage are present), yellow = moderately increased risk, orange = high risk, and red = very high risk.

**Table 6:** KDIGO heatmap categories among 16,387 women aged ≥75 years with a CKD diagnosis, based on the most recent eGFR and uACR values recorded between 2015–2019.

eGFR/uACR	A1	A2	A3	Total
G1	0 (0.00)	124 (0.76)	24 (0.15)	148 (0.90)
G2	0 (0.00)	2410 (14.71)	275 (1.68)	2685 (16.38)
G3a	4795 (29.26)	1897 (11.58)	216 (1.32)	6908 (42.16)
G3b	3277 (20.00)	1699 (10.37)	243 (1.48)	5219 (31.85)
G4	644 (3.93)	583 (3.56)	135 (0.82)	1362 (8.31)
G5	12 (0.07)	29 (0.18)	24 (0.15)	65 (0.40)
Total	8728 (53.26)	6742 (41.14)	917 (5.60)	16,387 (100)

Values in parentheses represent the relative frequency (%) with respect to the total population (*N* = 111,557). eGFR: estimated glomerular filtration rate; uACR: urine albumin-to-creatinine ratio. G1 (≥90 mL/min/1.73 m^2^), G2 (60–89), G3a (45–59), G3b (30–44), G4 (15–29), G5 (<15). A1 (<30 mg/g), A2 (30–300 mg/g), A3 (>300 mg/g). Cells are color-coded according to KDIGO risk categories: green = low risk (if no other markers of kidney damage are present), yellow = moderately increased risk, orange = high risk, and red = very high risk.

**Table 7:** Sex-specific distribution of crude mortality rates (per 1000 person-years) by CKD subgroup among the 111,557 individuals aged ≥75 years with complete CKD screening.

CKD Subgroup	Deaths	Person-Years	Incidence-Rate	95% CI
Men				
G1–G2	6256	101,031	61.92	60.40–63.48
G3a–G3b	3439	31,682	108.55	104.95–112.24
G4–G5	385	1513	254.46	229.68–281.19
Total	10,080	134,226	75.10	73.64–76.58
Women				
G1–G2	5912	142,978	41.35	40.30–42.42
G3a–G3b	4209	52,700	79.87	77.47–82.32
G4–G5	714	3626	196.91	182.73–211.90
Total	10,835	199,304	54.36	53.35–55.40

CKD: Chronic kidney disease.

**Table 8:** Sex- and age-specific distributions of crude mortality rates (per 1000 person-years) by CKD subgroup among the 111,557 individuals aged ≥75 years with complete CKD screening.

Age Group	CKD Subgroup	Deaths	Person-Years	Incidence-Rate	95% CI
Men					
75–84 y	G1–G2	3528	81,616	43.2	41.8–43.2
	G3a–G3b	1414	20,413	69.3	65.7–73.0
	G4–G5	138	749	184.2	154.8–217.7
	Total	5080	102,778	49.4	48.0–49.4
85–94 y	G1–G2	2589	19,010	136.2	131.0–141.5
	G3a–G3b	1864	10,816	172.3	164.6–180.3
	G4–G5	225	714	315.1	275.3–359.1
	Total	4678	30,540	153.2	148.8–153.2
≥95 y	G1–G2	139	405	343.2	288.5–405.2
	G3a–G3b	161	453	355.4	302.6–414.7
	G4–G5	22	50	440.0	275.7–666.2
	Total	322	908	354.6	316.9–395.5
Women					
75–84 y	G1–G2	2452	105,685	23.2	22.3–24.1
	G3a–G3b	1099	27,312	40.2	37.9–42.7
	G4–G5	144	1246	115.6	97.5–136.1
	Total	3695	134,243	27.5	26.6–27.5
85–94 y	G1–G2	3137	35,998	87.1	84.1–87.2
	G3a–G3b	2651	23,760	111.6	107.4–115.9
	G4–G5	477	2130	223.9	204.3–245.0
	Total	6265	61,888	101.2	98.7–101.3
≥95 y	G1–G2	323	1295	249.4	223.0–278.2
	G3a–G3b	459	1628	281.9	256.7–308.9
	G4–G5	93	250	372.0	300.3–455.7
	Total	875	3173	275.7	257.8–294.7

Incidence rates (per 1000 patient-years) and 95% confidence intervals were estimated assuming a Poisson distribution. CKD: Chronic kidney disease. 95% CI: 95% confidence interval.

**Table 9:** Cox regression analysis of all-cause mortality according to eGFR and albuminuria categories.

	HR	95% CI	*p*-Value
eGFR ≥ 60 mL/min/1.73 m^2^	1		
eGFR 45–59 mL/min/1.73 m^2^	1.142	1.103–1.182	<0.001
eGFR 30–44 mL/min/1.73 m^2^	1.419	1.361–1.480	<0.001
eGFR < 30 mL/min/1.73 m^2^	1.911	1.791–2.039	<0.001
No albuminuria	1		
Microalbuminuria	1.542	1.496–1.590	<0.001
Macroalbuminuria	1.870	1.749–2.000	<0.001

Model adjusted for sex, age, diabetes mellitus, hypertension, obesity, cancer, history of myocardial infarction, history of stroke, and use of antiplatelet agents, statins, renin–angiotensin–aldosterone system (RAAS) inhibitors, dipeptidyl peptidase-4 (DPP-4) inhibitors, and sodium–glucose cotransporter-2 (SGLT2) inhibitors. eGFR: estimated glomerular filtration rate. HR: hazard ratio. 95% CI: 95% confidence interval.

**Table 10:** Cox regression analysis of all-cause mortality according to KDIGO CKD risk categories.

	HR	95% CI	*p*-Value
Low Risk KDIGO	1		
Moderately Risk KDIGO	1.386	1.341–1.434	<0.001
High Risk KDIGO	1.742	1.672–1.814	<0.001
Very High Risk KDIGO	2.302	2.200–2.409	<0.001

Model adjusted for sex, age, diabetes mellitus, hypertension, obesity, cancer, history of myocardial infarction, history of stroke, and use of antiplatelet agents, statins, renin–angiotensin–aldosterone system (RAAS) inhibitors, dipeptidyl peptidase-4 (DPP-4) inhibitors, and sodium–glucose cotransporter-2 (SGLT2) inhibitors. eGFR: estimated glomerular filtration rate. HR: hazard ratio. 95% CI: 95% confidence interval.

**Table 11:** Cox regression model of risk of mortality according to eGFR and albuminuria categories.

eGFR (mL/min/1.73 m^2^)	ACR < 30 mg/g	ACR 30–299 mg/g	ACR > 300 mg/g
≥60 (reference)	1.0 (1.00–1.00)	1.68 (1.61–1.75)	2.32 (2.04–2.64)
45–59	1.24 (1.18–1.29)	1.86 (1.60–2.18)	2.24 (1.54–3.26)
30–44	1.49 (1.40–1.58)	2.24 (1.85–2.70)	2.72 (1.84–4.01)
<30	2.10 (1.81–2.42)	2.90 (2.01–4.17)	3.92 (2.28–6.75)

Model adjusted for sex, age, diabetes mellitus, hypertension, obesity, cancer, history of myocardial infarction, history of stroke, and use of antiplatelet agents, statins, renin–angiotensin–aldosterone system (RAAS) inhibitors, dipeptidyl peptidase-4 (DPP-4) inhibitors, and sodium–glucose cotransporter-2 (SGLT2) inhibitors. eGFR: estimated glomerular filtration rate. HR: hazard ratio. 95% CI: 95% confidence interval. ACR: albumin-to-creatinine ratio.

## Data Availability

The datasets generated and/or analysed during the current study are available from the corresponding author upon reasonable request.

## Supplementary Materials

The following supporting information can be downloaded at: https://www.mdpi.com/article/10.3390/jcm15166151/s1. Table S1. Distribution of CKD risk subgroups among the 45,943 men ≥ 75 years with complete CKD screening in the Community of Madrid; Table S2. Distribution of CKD risk subgroups among the 65,614 women ≥ 75 years with complete CKD screening in the Community of Madrid; Table S3. Sensitivity analysis: Cox regression of all-cause mortality; Table S4. Sensitivity analysis: Cox regression of all-cause mortality; Figure S1. Distribution of Pharmacologic Therapy (%), Complete CKD Screening Cohort; Figure S2. Kaplan–Meier survival curves according to eGFR Category, stratified by sex, over 40 months of follow-up; Figure S3. Kaplan–Meier survival curves according to albuminuria status stratified by sex over 40 months of follow-up; Figure S4. Kaplan–Meier survival curves according to KDIGO Risk Category stratified by sex over 40 months of follow-up.

## Abbreviations

AMI	Acute myocardial infarction
BMI	Body mass index
CEIMR	Regional Research Ethics Committee on Medicines of Madrid
CKD	Chronic kidney disease
CKD-EPI	Chronic Kidney Disease Epidemiology Collaboration
CMBD	Hospital discharge records
DPP-4	dipeptidyl peptidase-4
eGFR	Glomerular filtration rate
EMRs	Electronic medical records
GLP-1	Glucagon-like peptide 1
HT	Hypertension
INDEF	National Death Index of the Ministry of Health
PAD	Peripheral arterial disease
RAAS	Renin–angiotensin–aldosterone system
SD	Standard deviation
SGLT2	Sodium-Glucose Cotransporter-2
T2DM	Type 2 diabetes mellitus
uACR	Urine albumin-to-creatinine ratio
