# Supplementary material for: Implementation of KDIGO CKD Screening and Its Prognostic Implications in Community-Dwelling Adults Aged 75 Years or Older: A Population-Based Cohort Study
Source: J Clin Med. 2026 Aug 7;15(16):6151. doi: 10.3390/jcm15166151 (PMC13512940; doi:10.3390/jcm15166151)
Supplement: Supplementary file 1 [file jcm-15-06151-s001.zip › jcm-4460382-supplementary.pdf]

Supplementary Materials

Supplementary Table S1. *Distribution of CKD risk subgroups among the 45,943 men ≥ 75 years with complete CKD screening in the Community of Madrid.*

eGFR/UACR	A1	A2	A3	Total
G1	1,663 (3.62%)	350 (0.76%)	21 (0.05%)	2,034 (4.43%)
G2	25,001 (54.43%)	6,154 (13.40%)	585 (1.27%)	31,740 (69.10%)
G3a	5,464 (11.90%)	2,416 (5.26%)	360 (0.78%)	8,240 (17.94%)
G3b	1,651 (3.59%)	1,302 (2.83%)	289 (0.63%)	3,242 (7.06%)
G4	192 (0.42%)	330 (0.72%)	134 (0.29%)	656 (1.43%)
G5	1 (0.00%)	15 (0.03%)	15 (0.03%)	31 (0.07%)
Total	33,972 (73.96%)	10,567 (23.01%)	1,404 (3.06%)	45,943 (100%)

eGFR: estimated glomerular filtration rate; UACR: urine albumin-to-creatinine ratio. G1 (≥90 ml/min/1.73 m²), G2 (60–89), G3a (45–59), G3b (30–44), G4 (15–29), and G5 (<15). A1 (<30 mg/g), A2 (30–300 mg/g), and A3 (>300 mg/g). Cells are color-coded according to KDIGO risk categories: green = low risk (if no other markers of kidney damage are present), yellow = moderately increased risk, orange = high risk, and red = very high risk

Supplementary Table S2 *Distribution of CKD risk subgroups among the 65,614 women ≥ 75 years with complete CKD screening in the Community of Madrid.*

eGFR/UACR	A1	A2	A3	Total
G1	2,171 (3.31%)	327 (0.50%)	28 (0.04%)	2,526 (3.85%)
G2	36,925 (56.28%)	6,204 (9.46%)	409 (0.62%)	4,3538 (66.36%)
G3a	9,365 (14.27%)	2,544 (3.88%)	242 (0.37%)	12,151 (18.52%)
G3b	3,852 (5.87%)	1,803 (2.75%)	246 (0.38%)	5,901 (8.99%)
G4	702 (1.07%)	591 (0.90%)	138 (0.21%)	1,431 (2.18%)
G5	14 (0.02%)	29 (0.04%)	24 (0.04%)	67 (0.10%)
Total	53,029 (80.82%)	11,498 (17.52%)	1,087 (1.66%)	65,614 (100%)

eGFR: estimated glomerular filtration rate; UACR: urine albumin-to-creatinine ratio. G1 (≥90 ml/min/1.73 m²), G2 (60–89), G3a (45–59), G3b (30–44), G4 (15–29), and G5 (<15). A1 (<30 mg/g), A2 (30–300 mg/g), and A3 (>300 mg/g). Cells are color-coded according to KDIGO risk categories: green = low risk (if no other markers of kidney damage are present), yellow = moderately increased risk, orange = high risk, and red = very high risk.

Supplementary Table S3. *Sensitivity analysis: Cox regression of all-cause mortality.*

	OR	95% CI	p-value
eGFR >60 mL/min/1.73 m^2	1		
eGFR 45-59 mL/min/1.73 m^2	1.129	1.081-1.178	<0.001
eGFR 30-44 mL/min/1.73 m^2	1.414	1.342-1.490	<0.001
eGFR <30 mL/min/1.73 m^2	1.927	1.774-2.094	<0.001
No albuminuria	1		
Microalbuminuria	1.529	1.472-1.589	<0.001
Macroalbuminuria	1.908	1.753-2.077	<0.001

Model adjusted for age, sex, diabetes mellitus, hypertension, obesity, cancer, history of myocardial infarction, history of stroke, and use of antiplatelet agents, statins, renin–angiotensin–aldosterone system

(RAAS) inhibitors, dipeptidyl peptidase-4 (DPP-4) inhibitors, and sodium–glucose cotransporter-2 (SGLT2) inhibitors. eGFR: estimated glomerular filtration rate. OR: odds ratio.

Supplementary Table S4. *Sensitivity analysis: Cox regression of all-cause mortality.*

	**OR**	**95% CI**	**p-value**
Low Risk KDIGO	1		
Moderately Increased Risk KDIGO	1.361	1.306-1.418	<0.001
High Risk KDIGO	1.731	1.646-1.821	<0.001
Very High Risk KDIGO	2.247	2.122-2.380	<0.001

Model adjusted for age, sex, diabetes mellitus, hypertension, obesity, cancer, history of myocardial infarction, history of stroke, and use of antiplatelet agents, statins, renin–angiotensin–aldosterone system (RAAS) inhibitors, dipeptidyl peptidase-4 (DPP-4) inhibitors, and sodium–glucose cotransporter-2 (SGLT2) inhibitors. eGFR: estimated glomerular filtration rate. OR: odds ratio.

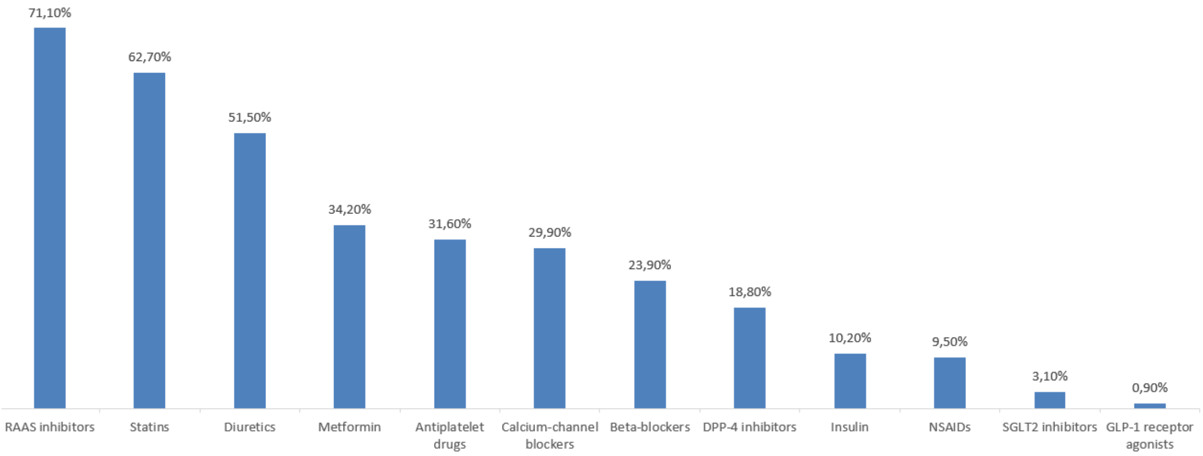

RAAS inhibitors: Renin–Angiotensin–Aldosterone System inhibitors. DPP-4 inhibitors: dipeptidyl peptidase-4 inhibitors. NSAIDs: Non-steroidal anti-inflammatory drugs. SGLT2 inhibitors: sodium-glucose co-transporter 2 inhibitors. GLP-1 receptor agonists: glucagon-like peptide-1 receptor agonists.

Supplementary Figure S1. *Distribution of Pharmacologic Therapy (%), Complete CKD Screening Cohort.*

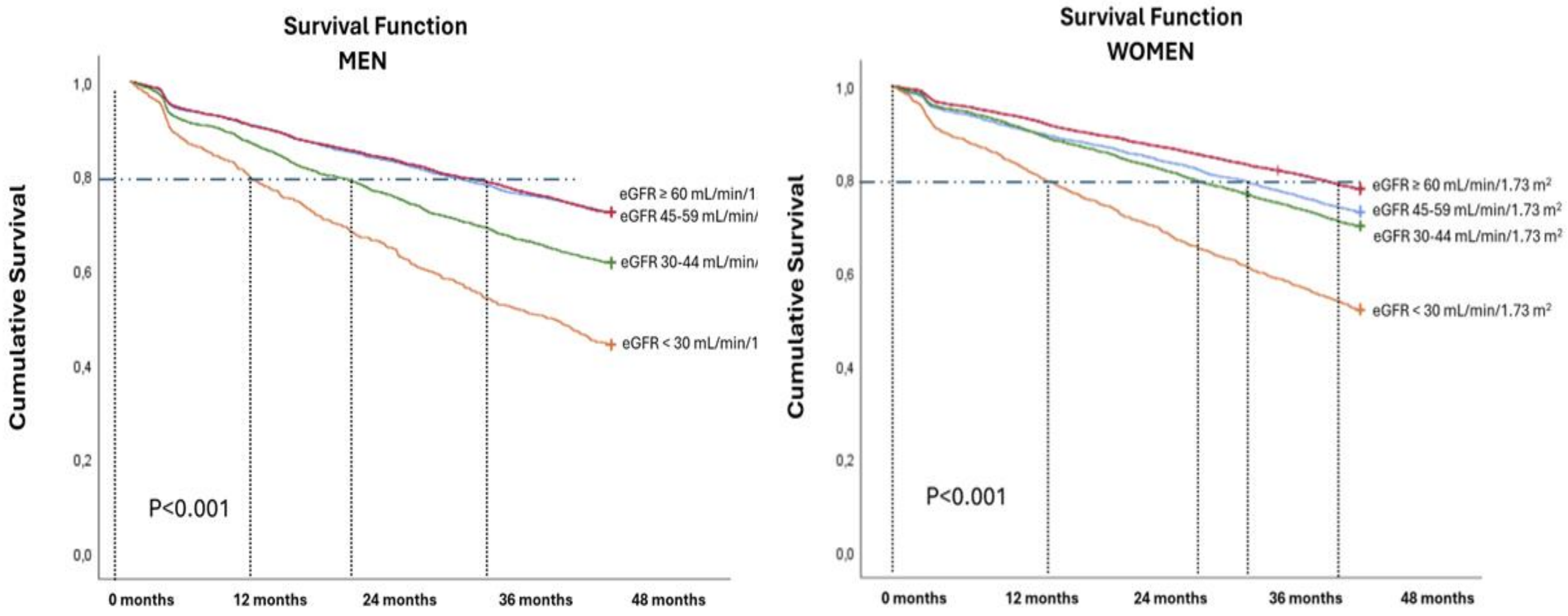

eGFR: estimated glomerular filtration rate.

Supplementary Figure S2. *Kaplan–Meier survival curves according to eGFR Category, stratified by sex, over 40 months of follow-up.*

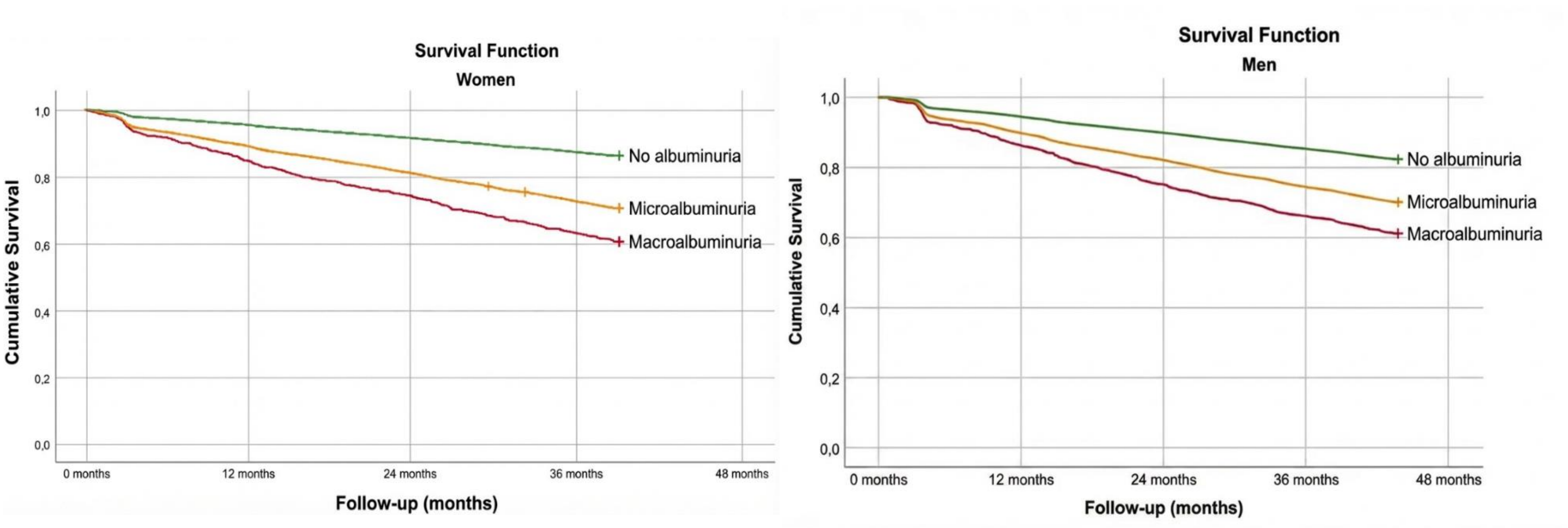

Note: Women with macroalbuminuria maintained a 75% survival probability slightly longer than men (~21 vs. ~19 months), and a similar 3–4-month advantage was observed for microalbuminuria.

Supplementary Figure S3. *Kaplan–Meier survival curves according to albuminuria status stratified by sex over 40 months of follow-up.*

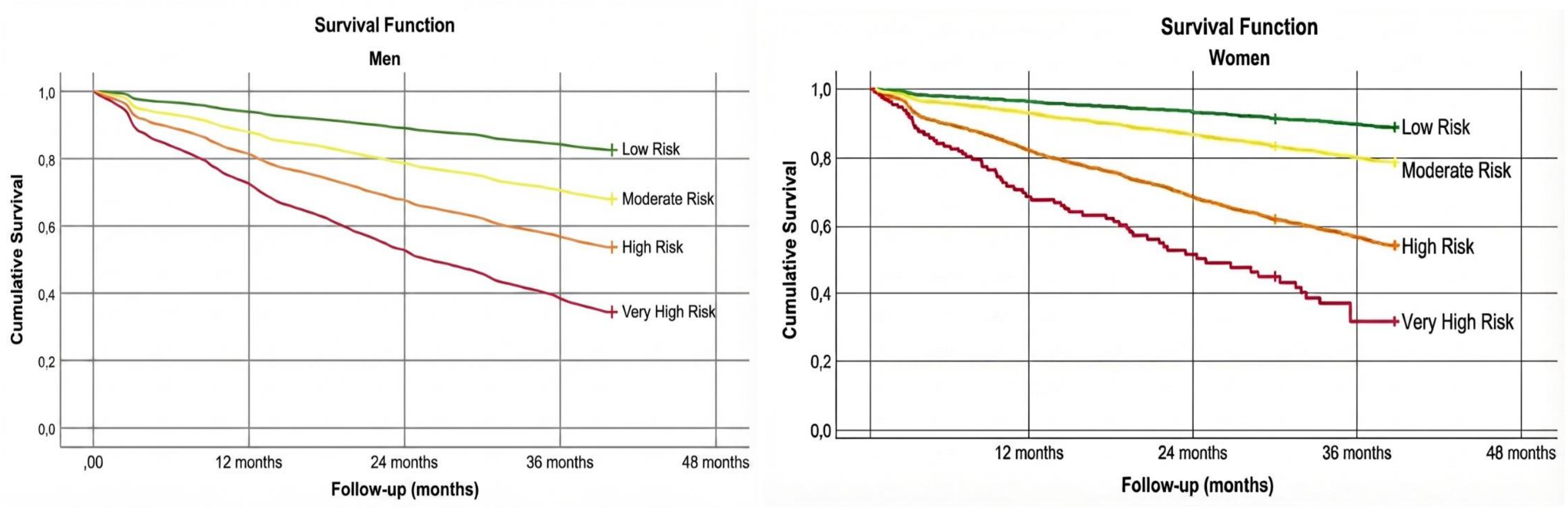

Supplementary Figure S4. *Kaplan–Meier survival curves according to KDIGO Risk Category stratified by sex over 40 months of follow-up.*

Supplementary Material – KDIGO Joint Risk Matrix Construction

---

---

title: "Supplementary Material – Construction of KDIGO Joint eGFR–ACR Risk Matrix"

output:

  html_document:

    toc: true

    toc_depth: 3

    number_sections: true

Introduction

This supplementary document describes the analytic procedure used to construct the joint KDIGO eGFR–ACR risk matrix presented in the manuscript. The approach is based on a Cox proportional hazards model including main effects for eGFR and ACR categories, as well as interaction terms between them. The model was fitted using R, and hazard ratios (HRs) with 95% confidence intervals (CIs) were computed for each eGFR × ACR combination.

All variable names have been translated into English for clarity and reproducibility.

1. Loading the SPSS Dataset

```r

library(haven)

library(dplyr)

library(survival)

# Load SPSS dataset

data <- read_sav("your_file.sav")

**2. Cox Model with Main Effects and Interaction Terms**

The model includes:
```

- eGFR categories:
  - eGFR_45_59
  - eGFR_30_44
  - eGFR_lt30
- ACR categories:
  - ACR_Micro
  - ACR_Macro
- Interaction terms:
  - eGFR45_ACRmicro, eGFR45_ACRmacro
  - eGFR30_ACRmicro, eGFR30_ACRmacro
  - eGFRlt30_ACRmicro, eGFRlt30_ACRmacro

```
cox_model <- coxph(
  Surv(Time_until_April2023, Death_2020_2023 == 1) ~
    eGFR_45_59 + eGFR_30_44 + eGFR_lt30 +
    ACR_Micro + ACR_Macro +
    eGFR45_ACRmicro + eGFR45_ACRmacro +
    eGFR30_ACRmicro + eGFR30_ACRmacro +
    eGFRlt30_ACRmicro + eGFRlt30_ACRmacro +
    Sex + Age + Diabetes + Hypertension +
    Myocardial_Infarction + Stroke + Obesity +
    Cancer + Aspirin + DPP4i + SGLT2i +
    Statin + RAAS_Blocker,
  data = data
)
```

3. Extracting Coefficients and Variance–Covariance Matrix

```
coefs <- coef(cox_model)
vcov_matrix <- vcov(cox_model)
```

4. Function to Compute HR and 95% CI for Any Combination

This function computes the HR and CI for any combination of coefficients, including interaction terms.

```
calc_hr_ci <- function(vars, coefs, vcov_matrix) {
  B <- sum(coefs[vars])
```

```
  V <- 0
  for (i in vars) {
    for (j in vars) {
      V <- V + vcov_matrix[i, j]
    }
  }

  SE <- sqrt(V)
  HR <- exp(B)
  lower <- exp(B - 1.96 * SE)
  upper <- exp(B + 1.96 * SE)

  return(c(HR = HR, LCI = lower, UCI = upper))
}
```

5. Computing Each eGFR × ACR Cell

Reference category:
eGFR ≥60 mL/min/1.73 m² and ACR <30 mg/g

```
# eGFR ≥60
cell_60_no    <- calc_hr_ci(c(), coefs, vcov_matrix)
cell_60_micro <- calc_hr_ci(c("ACR_Micro"), coefs, vcov_matrix)
cell_60_macro <- calc_hr_ci(c("ACR_Macro"), coefs, vcov_matrix)

# eGFR 45–59
cell_45_no    <- calc_hr_ci(c("eGFR_45_59"), coefs, vcov_matrix)
cell_45_micro <- calc_hr_ci(c("eGFR_45_59", "ACR_Micro", "eGFR45_ACRmicro"), coefs, vcov_matrix)
cell_45_macro <- calc_hr_ci(c("eGFR_45_59", "ACR_Macro", "eGFR45_ACRmacro"), coefs, vcov_matrix)

# eGFR 30–44
cell_30_no    <- calc_hr_ci(c("eGFR_30_44"), coefs, vcov_matrix)
cell_30_micro <- calc_hr_ci(c("eGFR_30_44", "ACR_Micro", "eGFR30_ACRmicro"), coefs, vcov_matrix)
```

```r
cell_30_macro <- calc_hr_ci(c("eGFR_30_44", "ACR_Macro", "eGFR30_ACRmacro"), coefs, vcov_matrix)


# eGFR <30

cell_lt30_no    <- calc_hr_ci(c("eGFR_lt30"), coefs, vcov_matrix)

cell_lt30_micro <- calc_hr_ci(c("eGFR_lt30", "ACR_Micro", "eGFRlt30_ACRmicro"), coefs, vcov_matrix)

cell_lt30_macro <- calc_hr_ci(c("eGFR_lt30", "ACR_Macro", "eGFRlt30_ACRmacro"), coefs, vcov_matrix)
```

6. Final KDIGO Joint Risk Table

```r
table_joint <- data.frame(

  eGFR = c("≥60", "45–59", "30–44", "<30"),

  ACR_No    = rbind(cell_60_no, cell_45_no, cell_30_no, cell_lt30_no),

  ACR_Micro = rbind(cell_60_micro, cell_45_micro, cell_30_micro, cell_lt30_micro),

  ACR_Macro = rbind(cell_60_macro, cell_45_macro, cell_30_macro, cell_lt30_macro)

)


table_joint
```

7. Interpretation

This table provides hazard ratios and 95% confidence intervals for each eGFR × ACR combination, fully aligned with KDIGO risk stratification. The approach allows quantification of synergistic effects between filtration decline and albuminuria, complementing the independent associations estimated in the main Cox models.
